# Surveillance of avirulent Newcastle disease viruses at live bird markets in Eastern China during 2008–2012 reveals a new sub-genotype of class I virus

**DOI:** 10.1186/s12985-014-0211-2

**Published:** 2014-12-04

**Authors:** Jie Zhu, Haixu Xu, Jingjing Liu, Zhenzhen Zhao, Shunlin Hu, Xiaoquan Wang, Xiufan Liu

**Affiliations:** Animal Infectious Disease Laboratory, College of Veterinary Medicine, Yangzhou University, 12 East Wenhui Road, Yangzhou, Jiangsu 225009 China; Jiangsu Co-innovation Center for Prevention and Control of Important Animal Infectious Diseases and Zoonoses, Yangzhou University, Yangzhou, 225009 China

**Keywords:** Class I Newcastle disease virus, Sub-genotype 3c, Sub-genotype 1b, Genetic, Antigenticy

## Abstract

**Background:**

The strains of Newcastle disease virus (NDV) can be divided into two distinct clades: class I and class II. At present, limited molecular epidemiological data are available for the class I virus at live bird markets (LBMs). Knowing the genomic and antigenic characteristics of class I NDVs might provide important insights into the evolution dynamics of these viruses. In this study class I NDVs isolated from LBMs in Eastern China between 2008 and 2012 were characterized.

**Results:**

We characterized 34 class I NDVs genetically and 15 of the 34 NDVs pathologically which originated from geese, chickens and ducks at live bird markets. Based on the older classification system, twelve of fourteen strains isolated from 2008 to 2010 belonged to sub-genotype 3b. However, the rest 22 strains formed a separate novel cluster in genotype 3, which was designated as sub-genotype 3c. When based on the new classification system, sub-genotype 3b was classified into sub-genotype 1a and the sub-genotype 3c was classified into sub-genotype 1b. Over 62% (21/34) of the viruses were chicken-origin and only 13 isolates were waterfowl-origin. The Cross-neutralization reactions between CK/JS/05/11, CK/JS/06/12 and the vaccine strain LaSota showed significant antigenic differences between them.

**Conclusions:**

Currently, sub-genotype 3c (or 1b) NDVs are the most frequently isolated classI strains at LBMs in Eastern China., and the class I NDVs has transferred from waterfowls to chickens and circulated in chicken flocks extensively.

## Background

Newcastle disease (ND) is caused by Newcastle disease virus(NDV)which has caused severe economic losses all around the world [1,2]. NDV is a member of avian paramyxovirus (APMV), which belongs to the genus Avulavirus, sub-family *Paramyxovirinae*, family *Paramyxoviridae*, and order *Mononegavirales* [3]. The genome of NDV is a single-stranded, negative-sense, nonsegmented RNA of approximately 15.2 kb. The viral genome is comprised of six genes: nucleocapsid (NP), phosphoprotein (P), matrix (M), fusion (F), hemagglutinin–neuraminidase (HN), and RNA-dependent RNA polymerase (L) [4]. The strains of NDV can be divided into two distinct clades: class I and class II, and based on the older classification system, both clades could be divided into 9 genotypes, and the class II clade can be divided into fifteen genotypes when based on the new classification system, while the class I clade can be divided into three sub-genotypes [5–7].

Liu et.al has found that the class I virus strains circulating among Eastern China were genotype 2 and 3, while the genotype 3 can be divided into two sub-genotype: 3a and 3b [8]. In 2012, one class I virus isolated in Eastern China was ascribed to genotype 4 [9], which means that the class I viruses circulating in Eastern China were mix. As Liu et al. [8] only concerned about the class I virus circulating in ducks at LBMs in Eastern China, and deals of chickens were also existed at the live bird markets (LBMs), however, the circulation of the class I virus among chickens has not been well elucidated. In this study, we collected 7106 cloacal swabs from 600 flocks including both chickens and waterfowls for virus isolation from 2008 to 2012 and 34 class I isolates were characterized phylogentically while and 15 of the 34 NDVs were pathologically characterized.

## Results

### Isolation and identification of the NDVs from the LBMs in Eastern China

We obtained a total of 7106 samples by monthly collections from multiple avian species at LBMs in Eastern China during May 2008 to April 2012, and 247 NDVs were isolated and identified with a total isolation rate of 3.48%. As is shown in Table 1, a total of 3.32% (236/7106) class I NDVs were identified and the isolation rates in colder months (from November to March) were higher than in warmer months (from April to October) which is consistent with the previous report [8]. Furthermore, the monthly isolation rate varied from 0 to 7.5% and the yearly isolation rate varied from 2.91% to 5.67% and January 2012 had the highest isolation rate although no specific thing about these times was found. The 236 class I NDVs were collected from 34 flocks (21 from the chickens and 13 from the waterfowls), and the details for these isolates were listed in Table 2.

**Table 1:** **Monthly isolations of NDVs from cloacal swabs of multiple avian species at live bird markets for wholesale in Eastern China during May 2008 to April 2012**

**Year**	**Positive/sample**												**Total**	**Isolation rate (%)**
	**Jan**	**Feb**	**Mar**	**Apr**	**May**	**June**	**July**	**Aug**	**Sep**	**Oct**	**Nov**	**Dec**		
2008	-	-	-	-	4/200	8/180	1/90	1/100	10/200	0/100	4/100	4/130	32/1100	2.91
2009	8/200	8/180	5/100	5/140	1/100	5/130	0/130	1/160	2/130	7/200	4/110	5/120	51/1700	3.00
2010	9/160	7/150	11/180	5/140	1/100	5/130	0/140	1/140	2/180	7/190	4/150	5/140	57/1800	3.16
2011	11/180	5/160	8/180	7/140	5/100	2/130	0/150	0/130	4/150	5/160	2/175	7/145	56/1800	3.11
2012	15/200	13/180	10/150	10/176	-	-	-	-	-	-	-	-	40/706	5.67
total	43/740	33/670	34/610	27/596	11/500	20/570	1/510	3/530	18/660	19/650	14/535	21/535		
Isolation Rate (%)	5.81	4.93	5.57	4.53	2.20	3.51	0.200	0.57	2.73	2.92	2.62	3.93		3.32

**Table 2:** **Details of the representative NDVs isolated from the domestic avian species at LBMs in Eastern China during 2008 to 2012**

**Virus strains**	**Year of isolation**	**Location (Province)**	**Host**	**ICPI**	**MDT**	**Cleavage site of fusion protein**	**Class**	**subgenotype**	**Accession number**
D/JS/1/08	2008	Jiangsu	Duck	0	>120 h	EQQERL	I	3b	KM509016
CK/AH/02/08	2008	Anhui	Chicken	0	>120 h	EQQERL	I	3b	KM509017
Go/SD/03/08	2008	Shandong	Goose	0	>120 h	EQQERL	I	3b	KM509018
CK/HeN/04/08	2008	Henan	Chicken	NT^a^	NT	EQQERL	I	3b	KM509019
Go/JS/01/09	2009	Jiangsu	Goose	0	>120 h	EQQGRL	I	3b	KM509020
Go/JS/02/09	2009	Jiangsu	Goose	0	>120 h	EQQERL	I	3b	KM509021
D/JS/03/09	2009	Jiangsu	Duck	0	>120 h	ERQERL	I	3c	KM509022
D/JS/04/09	2009	Jiangsu	Duck	NT	NT	EQQERL	I	3b	KM509023
CK/JS/05/09	2009	Jiangsu	Chicken	NT	NT	EQQERL	I	3b	KM509024
CK/SD/06/09	2009	Shandong	Chicken	NT	NT	EQQERL	I	3b	KM509025
D/JS/01/10	2010	Jiangsu	Duck	0	>120 h	EQQERL	I	3b	KM509026
CK/JS/02/10	2010	Jiangsu	Chicken	0	>120 h	ERQERL	I	3c	KM509027
CK/JS/03/10	2010	Jiangsu	Chicken	0	>120 h	EQQERL	I	3b	KM509028
CK/JS/04/10	2010	Jiangsu	Chicken	NT	NT	EQQERL	I	3b	KM509029
D/SH/05/10	2010	Shanghai	Duck	NT	NT	EQQERL	I	3b	KM509030
CK/JS/06/10	2010	Jiangsu	Chicken	NT	NT	ERQERL	I	3b	KM509031
Go/JS/01/11	2011	Jiangsu	Gose	0	>120 h	ERQERL	I	3c	KM509032
CK/JS/02/11	2011	Jiangsu	Chicken	0	>120 h	ERQERL	I	3c	KM509033
CK/JS/03/11	2011	Jiangsu	Chicken	0	>120 h	ERQERL	I	3c	KM509034
CK/JS/04/11	2011	Jiangsu	Chicken	NT	NT	ERQERL	I	3c	KM509035
CK/JS/05/11	2011	Jiangsu	Chicken	NT	NT	ERQERL	I	3c	KM509036
CK/JS/06/11	2011	Jiangsu	Chicken	NT	NT	ERQERL	I	3c	KM509037
CK/JS/07/11	2011	Jiangsu	Chicken	NT	NT	ERQERL	I	3c	KM509038
CK/JS/08/11	2011	Jiangsu	Chicken	NT	NT	ERQERL	I	3c	KM509039
D/JS/01/12	2012	Jiangsu	Duck	NT	NT	ERQERL	I	3c	KM509040
D/JS/02/12	2012	Jiangsu	Duck	0	>120 h	ERQERL	I	3c	KM509041
CK/JS/03/12	2012	Jiangsu	Chicken	0	>120 h	ERQERL	I	3c	KM509042
CK/JS/04/12	2012	Jiangsu	Chicken	0	>120 h	ERQERL	I	3c	KM509043
CK/JS/05/12	2012	Jiangsu	Chicken	NT	NT	ERQERL	I	3c	KM509044
CK/JS/06/12	2012	Jiangsu	Chicken	NT	NT	ERQERL	I	3c	KM509045
Go/JS/07/12	2012	Jiangsu	Goose	NT	NT	ERQERL	I	3c	KM509046
Go/SH/08/12	2012	Shanghai	Goose	NT	NT	ERQERL	I	3c	KM509047
CK/HeN/09/12	2012	Henan	Chicken	NT	NT	ERQERL	I	3c	KM509048
CK/SD/10/12	2012	Shandong	Chicken	NT	NT	ERQERL	I	3c	KM509049

^a^NT: not test.

### Phylogenetic analysis

**Figure 1:**
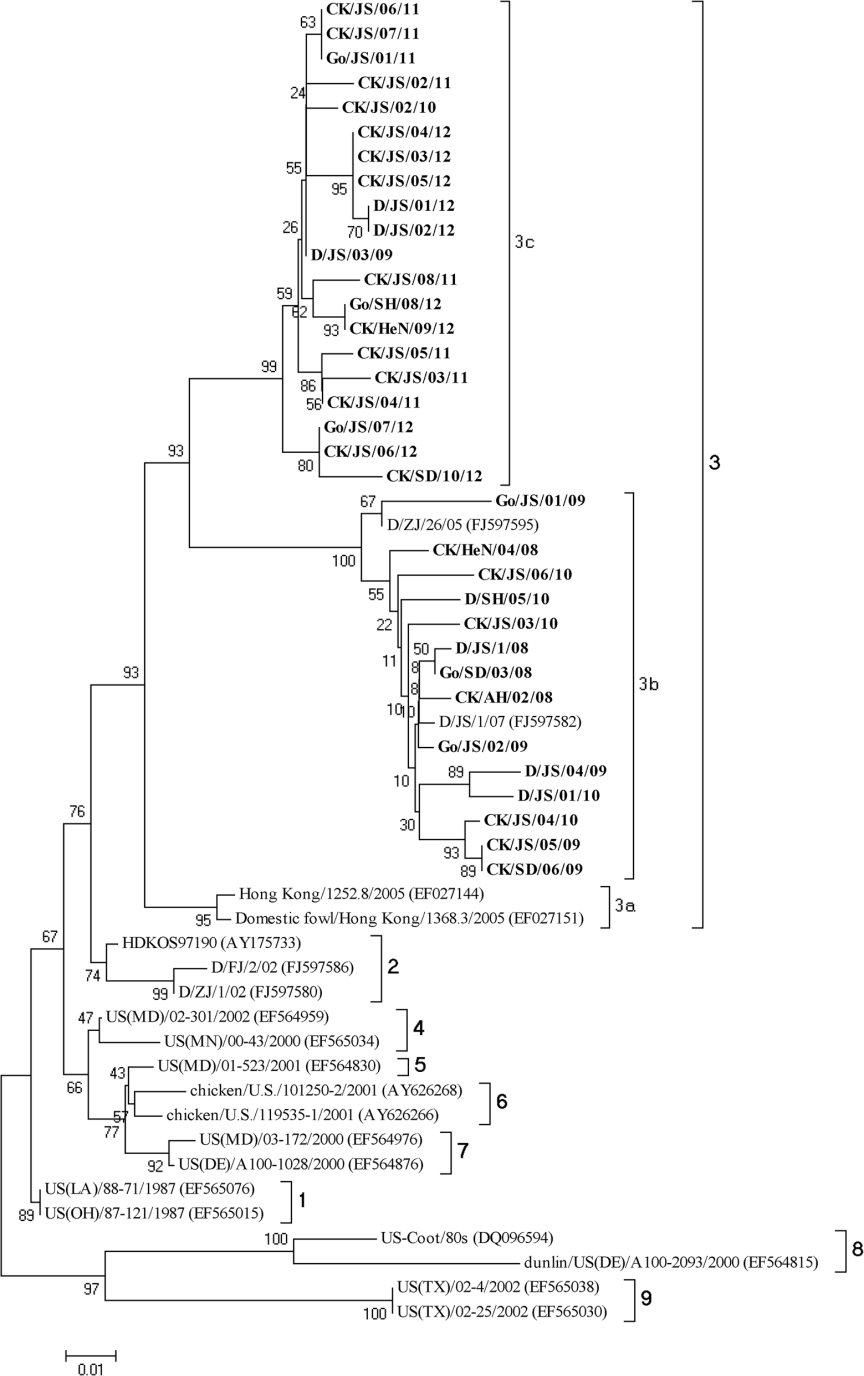
**Phylogenetic tree of class I NDV isolatess based on the older classification methods by the variable region (nt 47–420) of the F gene.** The reference class I NDV isolates characterized in this study were shown in bold.

236 samples were detected to be positive in the 7106 swabs, but the result of sequences showed that the viruses isolated in the same flock were 100% homology. At last, we have got 34 isolates in this manuscript. The F gene of the 34 NDVs from each flock were sequenced and analyzed phylogentically with 20 representative reference class I F gene sequences, and the phylogentic tree was constructed based on the variable region of the F genes (47-420 nt) which was shown in Figure 1.

**Figure 2:**
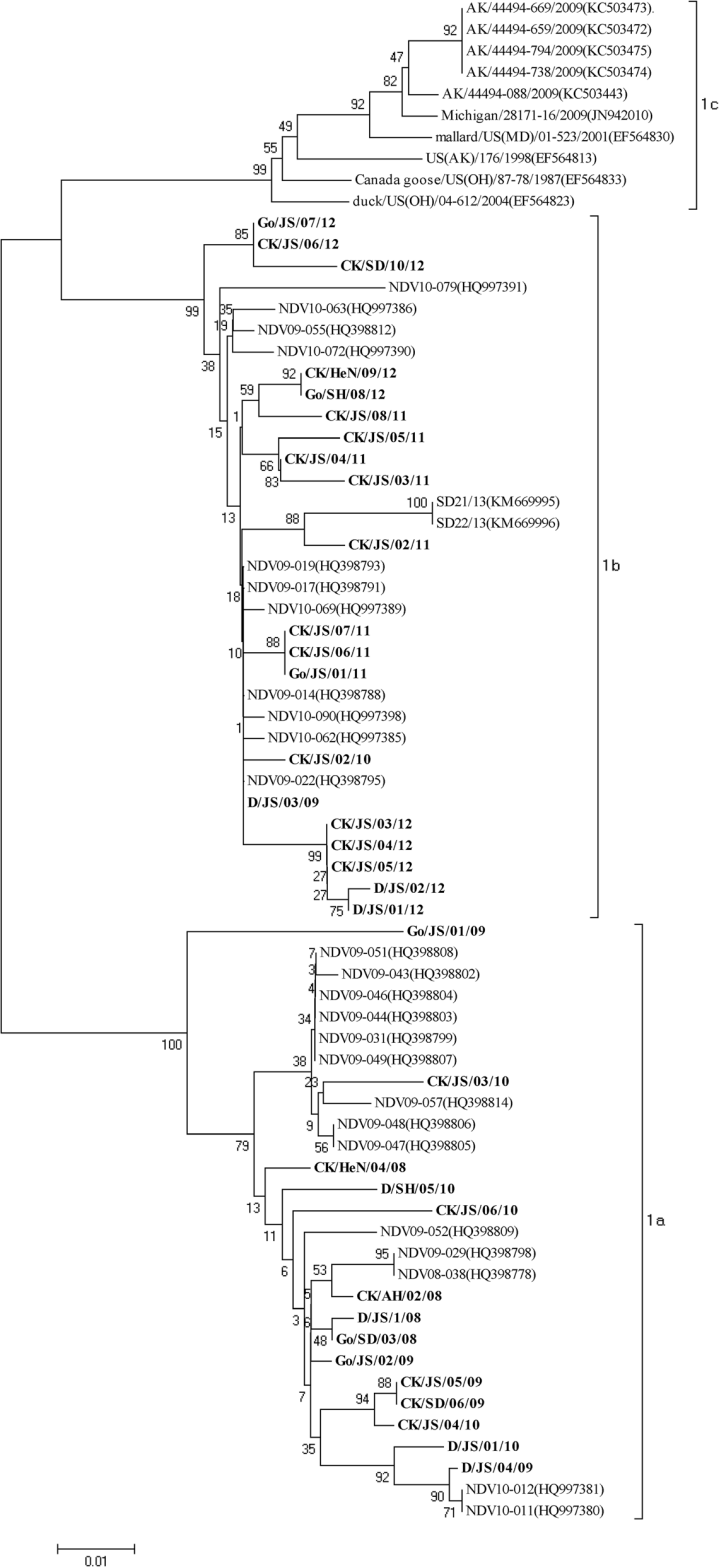
**Phylogenetic tree of class I NDV isolatess based on the new classification methods by the complete sequence the F genes.** The reference class I NDV isolates characterized in this study were shown in bold.

Based on the previous classification system, all the viruses were grouped together with the Hong Kong LBM strains and the earlier Eastern China strains which have been designated as genotype 3 [5,6,8]. Except CK/JS/02/10 and D/JS/03/09, all strains isolated among 2008 to 2010 were grouped together with two duck-origin virus isolated in Jiangsu and Zhejiang (D/ZJ/26/05, D/JS/1/07) that have been designated as sub-genotype 3b [8]. However, the rest 22strains formed a new cluster which evolutionary distance with sub-genotype 3a and 3b was 0.068 and 0.099 (Table 3), respectively. Therefore, this new cluster was separated from the two established sub-genotypes in genotype 3 and designated as sub-genotype 3c. As is shown in Figure 2, when utilized the new classification system proposed by Diel et al. [7], the 34 strains could also be clustered into two separated sub-genotypes: 1a and 1b. Meanwhile, the sub-genotype 3b belonged to sub-genotype 1a in the new classification system and sub-genotype 3c belonged to sub-genotype 1b.

**Table 3:** **Estimation of evolutionary distances between sub-genotypes of genotype 3**

**Sub-genotype**	**Evolutionary distances**		
	**3a**	**3b**	**3c**
3a		0.021	0.016
3b	0.101		0.020
3c	0.068	0.099

The above genotypic classification of NDVs could be also reflected by comparisons of the deduced amino acid sequence of the F gene. When deduced amino acid sequences of the F gene of the three sub-genotypes were compared, different amino acid profiles were present between the 3c and the other 2 sub-genotype NDVs in genotype 3. Three different amino acids were present between the 3a and 3c viruses of F gene (Table 4), and 3c strains displayed the substitutions W10L, E47D and V118I. As shown in Table 4, when compared with the sub-genotype 3b viruses, the 3c viruses had the following amino acid substitutions: P6S, V19A, S36P, R48K, I69M, Q113R, I165V, T176A, I206V, F214L, S231L, V237I, Y337H, I386L, L396M, R445Q, D489N, T522A, R530K and S531A.

**Table 4:** **Subtype-specific residue substitutions in the deduced genotype 3 F protein sequence**

**Sub-genotypes**	**6**	**10**	**12**	**18**	**19**	**22**	**36**	**47**	**48**	**69**	**113**	**118**	**165**	**176**	**206**	**214**	**231**	**237**	**337**	**386**	**396**	**445**	**489**	**522**	**530**	**531**
	**S**	**L**	**A**	**V**	**A**	**S**	**P**	**D**	**K**	**M**	**R**	**I**	**V**	**A**	**V**	**L**	**L**	**i**	**H**	**L**	**M**	**Q**	**N**	**A**	**K**	**A**
3a	-^a^	W	V	-	-	L/-	-	E	-	-	-	-	NT^b^	NT	NT	NT	NT	NT	NT	NT	NT	NT	NT	NT	NT	NT
3b	P/-	-	T/-	M	V	-	S/-	-	R	I	Q	-	I	T	I	F	S	V	Y	I	L	R	D	T	R	S
3c	-	-	V/-	L/-	T/-	-	Q/-	-	-	-	-	V	L/-	-	G/-	-	-	-	-	-	-	-	-	-	-	-

^a^-indicates genetic identity with the consensus residue.

^b^NT: not test.

### Pathogenicity tests

We selected 3 viruses each year to test the pathogenicity, and the results of the pathogenicity tests showed that all the selected isolates had an intracerebral pathogenicity index value 0 and a mean death time > 120 h, which was consistent with the tyipical avirulent motif ^112^EQ/RQERL^117^ at the F cleavage sites.

### Antigenic difference assays

As is shown in Table 5, cross-neutralization and -hemagglutination inhibition reactions between the two isolates confirmed the significant antigenic differences between the isolates and vaccine strain. In the neralization test, the R value between the CK/JS/05/11 and CK/JS/06/12 and LaSota were only 0.17 and 0.12, respectively. When 0.67 ≤ *R ≤* 1.5, indicates no significant antigenic difference between the two viruses and 0.5 ≤ *R* ≤ 0.67 indicates a minor difference between the two viruses. An R value of *R* < 0.5 indicates a major difference between the two virus strains.

**Table 5:** **Coefficients of antigenic similarity (R) between NDV isolates and LaSota strain**

**Strain**	**LaSota**
CK/JS/05/11	0.24^a^ 0.17^b^
CK/JS/06/12	0.27^a^ 0.12^b^

^a^Cross-hemagglutination inhibition test.

^b^Chicken embryo cross-neutralization test.

## Discussion

Currently, most of the avirulent NDVs isolated from the LBMs in America and Korea belonged to class I clade. [10–12]. Liu et.al have reported that the class I viruses circulated in waterfowls from 2002 to 2007 at LBMs in Eastern China were genotype 2 and 3 which could be divided into two separated groups: 3a and 3b [8]. In this paper, we conducted the continuous surveillance and characterization of class I clade NDVs both from the chicken and waterfowl flocks at LBMs in Eastern China during 2008 to 2012 and found a new separate cluster in genotype 3 which possessed its own characteristic amino acid substitutions on the F protein (Tables 1 and 2).

During May 2008 to April 2012, 247 NDVs were isolated from the LBMs in Eastern China and 95.5% (236/247) were class I NDVs, which indicated that the class I NDVs were still frequently isolated among the birds at LBMs. Researchers have already ensured that the class I clade NDVs mainly circulated among waterfowls [5,6,8], however in this research, 21 of the 34 classI NDV-positive flocks were the chicken-origin, which indicates that the cross-species transmission has been appeared and class I NDVs have already become frequently isolated among the chickens. In china, deals of chickens and waterfowls are made together at the LBMs, therefore the LBM has been one of the important sites for pathogens inter- or cross-species transmission. Considering the higher isolation of the class I NDVs at the LBMs, much attention should be paid to the transmission of class I NDV at this site.

All the isolates in this study were clustered into genotype 3 which was divided into two sub-genotypes 3a and 3b in the previous study [6,8]. However, a separate cluster was identified here based on the phylogenetic analysis of the partial F genes. As is illustrated by Diel et al. [7], different genotypes should have an average distance per site >10% (0.1) and different sub-genotypes should have an average distance per site between 3 (0.03) and 10% (0.1), while its evolutionary distance with 3a and 3b was 0.068 and 0.099, respectively, which has met the requirement to form a new sub-genotype and was designated here as 3c. Based on the two classification methods, the isolates from 2008–2010 are more similar to older previously studied isolates and the more recent strains (2011–2012) are clustering together and the waterfowl-origin isolation rate is higher than the 2011–2012 isolation rate, which means that the class I NDV is keeping evolution in the poultry flocks and can affect the chickens more efficiency. Comparing the partial F genes between the three sub-genotypes, we found that the sub-genotype 3a and 3c shared the same cleavage site EQQERL and 3c viruses showed both higher nucleotide and amino acid homologous with sub-genotype 3a when compared with 3b. Therefore the sub-genotype 3c viruses might evolve from the sub-genotype 3a.

Previously, seven neutralizing epitopes positioned at residues 72, 74, 75, 78, 79, 157 to 171, and 343 of the F protein have been identified [13–16]. When compared the F genes of 3c cluster with the vaccine strain LaSota, four of these epitopes were changed as follows: A75Q, A79S, V168I, and D170S, which displayed the antigenic difference between them and was confirmed by the results derived from the cross-HI and -neutralization tests in this study. Class I NDVs are the avirulent strains usually isolated from waterfowls [5,8], however, Yu et. al found that the class I avirulent virus could be generated into virulent one through consecutive passages in the chicken only by two residues alteration at the F cleavage site [17]. As is reported that, the virulent class I viruses which were phylogenetically closely related to those found in WS have previously been found [18,19], and the risk of ND epidemic would be introduced due to its high infection rate in the poultry flocks, the significant antigenic difference and low genetic homology with the vaccine strain [20]. Therefore, continuous surveillance for the class I NDVs both from the LBMs and poultry farms should be performed and strict control strategies should be developed to eliminate the potential risk caused by these NDVs.

## Conclusions

The NDVs isolated at LBMs during 2008–2012 belonged to sub-genotype 3b (or 1a), and a newly identified cluster 3c (or 1b) which has already transferred from waterfowls to terrestrial birds and has been frequently isolated in the chicken flocks extensively.

## Materials and methods

### Virus isolation and identification

**Figure 3:**
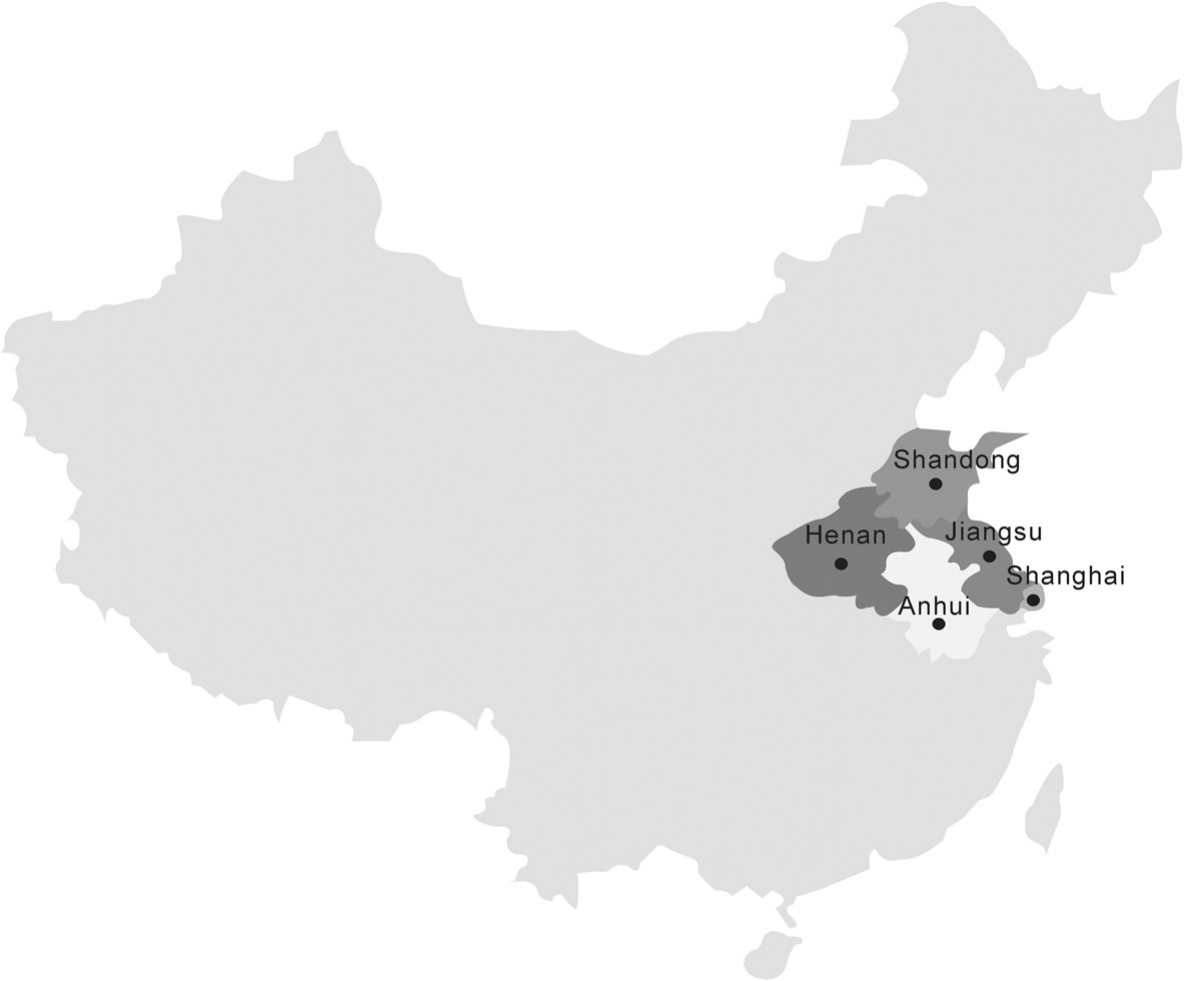
**Map of China showing Shanghai and Shandong, Henan, Anhui, Jiangsu provinces where sampling birds came from during the NDV surveillance program at LBMs.**

We collected cloacal swab samples from the LBMs in Eastern China (Figure 3) since 2008 to 2012, and each flocks we collected 5–15 samples. And all the swabs were inoculated into the allantoic cavities of 9 to 11 day old specific-pathogen-free (SPF) chicken embryos and incubated at 37°C for two passages of 96 hours each [6]. Allantoic fluids from the incubated eggs were harvested after the completion of two passages and were then assayed for hemagglutination activity (HA) by following the OIE protocols for NDV; the presence of NDV was confirmed by hemagglutination inhibition (HI) assay with NDV-specific polyclonal antiserum [21]. The NDV isolates obtained from the surveillance that were selected for pathogenicity tests and genetic studies are presented in Table 2.

### Viral RNA exaction

Virus RNA extraction was prepared from infective allantoic fluid using TRIzol LS (Invitrogen, Carlsbad, California, USA) according to the manufacturer’s instructions. The RNA pellets were resuspended in RNase-free water after washing with 70% ethanol and were stored at −70°C until use. And the initial RT reaction was performed as described previously [22].

### Sequencing of the partial F genes

According to the published F gene sequences from the GenBank database, we designed a pair of primers for the amplification of the partial F genes: FTY1: CGTAGAAAA AACACGGGTAGAAGA (nt 4494–4517), FTY2: CAGGTAGGTRGCACGCATATTATT (5429–5452). The DNA amplification was performed for 25 cycles consisting of 30 seconds at 94°C, 1 min at 58°C, and followed by a final elongation of 10 min at 72°C. The complete F genes were amplified by the primes designed by Liu et al. [8]. The PCR products were sequenced at Sangon (Shanghai, China).

### Phylogenetic analysis and inference of the evolutionary distances

In this study, we obtained class I reference NDV F gene sequences representing previously class I genotypes 1 to 9 based on the previously established classification system and reference strains representing the newly established classification system as 1a, 1b and 1c from the Genbank, while the accession numbers of these NDVs were shown in the phylogenetic trees. The prediction of amino acid sequences, alignment of sequences, and phylogenetic analysis were performed using MegAlign in the Lasergene package (DNASTAR Inc. Madison, WI, U.S.). Phylogenetic tree was created using the neighbor-joining method and MEGA 5.2 based on comparison of partial F gene sequences (between nt 47 and 420). The evolutionary distances were inferred by the software MEGA5 and the mean interpopulational evolutionary diversity (mean evolutionary distance between genotypes) was determined using the Maximum Composite Likelihood model [7,23].

### Pathogenicity tests

The intracerebral pathogenicity index in 1-day-old chicks and the mean death time in 9 to 11-day-old SPF chicken embryos were determined for some of the isolates in this study as Liu did previously [2].

### Comparision of the antigenic difference between the isolates and vaccine strain

The anti-serum against CK/JS/06/12, CK/JS/05/11 and LaSota were prepared from the SPF chickens vaccinated with inactivated oil-emulsion LaSota, CK/JS/06/12 and CK/JS/05/11, respectively. Three weeks post-vaccination, serum were collected and stored at −70°C until use. The coss-haemagglutination inhibition (HI) and cross-neutralization tests to measure the antigenic difference between the isolates and the vaccination strain LaSota, were performed as described previously [24].
